# Supplementary material for: Splice-Junction-Based Mapping of Alternative Isoforms in the Human Proteome
Source: Cell Rep. Author manuscript; Available in PMC 2020 Jan 15. (PMC6961840; doi:10.1016/j.celrep.2019.11.026)
Supplement: 2 [file NIHMS1546469-supplement-2.zip › DF1/PXD006675/InferiorVenaCava/InferiorVenaCava_3_SYPL1_ETSLHSPSNTSAPHSQGGIPPPTGL.pdf]

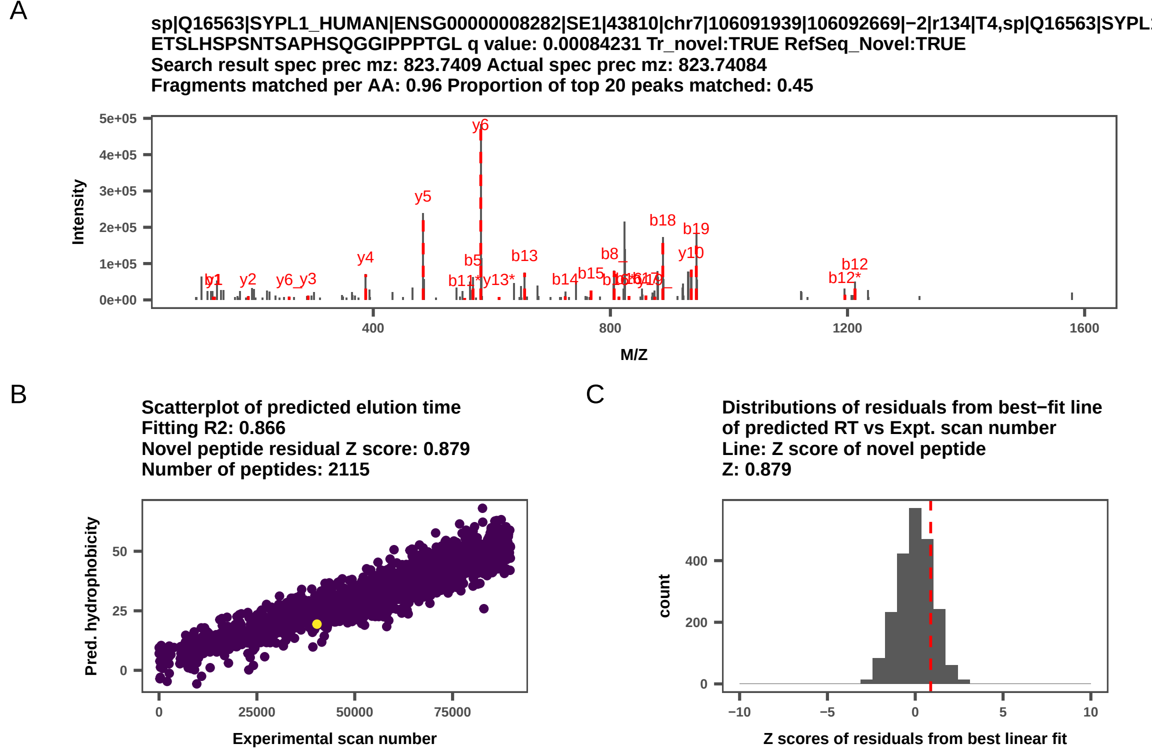
A
sp|Q16563|SYPL1_HUMAN|ENSG00000008282|SE1|43810|chr7|106091939|106092669|−2|r134|T4,sp|Q16563|SYPL
ETSLHSPSNTSAPHSQGGIPPPTGL q value: 0.00084231 Tr_novel:TRUE RefSeq_Novel:TRUE
Search result spec prec mz: 823.7409 Actual spec prec mz: 823.74084
Fragments matched per AA: 0.96 Proportion of top 20 peaks matched: 0.45
Intensity
5e+05
4e+05
3e+05
2e+05
1e+05
0e+00
y6
y5
b18
b19
y10
b13
b8_
y4
b5
b12
b12*
b11*
y13*
b14
b15
y2
y6_y3
400
800
1200
1600
M/Z
B
Scatterplot of predicted elution time
Fitting R2: 0.866
Novel peptide residual Z score: 0.879
Number of peptides: 2115
Pred. hydrophobicity
50
25
0
0
25000
50000
75000
Experimental scan number
C
Distributions of residuals from best−fit line
of predicted RT vs Expt. scan number
Line: Z score of novel peptide
Z: 0.879
count
400
200
0
−10
−5
0
5
10
Z scores of residuals from best linear fit
